# Tracking adoptive T cell immunotherapy using magnetic particle imaging

**DOI:** 10.7150/ntno.55165

**Published:** 2021-04-27

**Authors:** Angelie Rivera-Rodriguez, Lan B. Hoang-Minh, Andreina Chiu-Lam, Nicole Sarna, Leyda Marrero-Morales, Duane A. Mitchell, Carlos M. Rinaldi-Ramos

**Affiliations:** 1 J Crayton Pruitt Family Department of Biomedical Engineering, University of Florida, Gainesville, FL USA.; 2 Preston A. Wells, Jr. Center for Brain Tumor Therapy, University of Florida, Gainesville, FL USA.; 3 Lillian S. Wells Department of Neurosurgery, McKnight Brain Institute, University of Florida, Gainesville, FL USA.; 4 Department of Chemical Engineering, University of Florida, Gainesville, FL USA.; 5 UF Health Cancer Center, University of Florida, Gainesville, FL USA.

**Keywords:** biomedical imaging, brain cancer, cell labeling, cellular therapy, iron oxide nanoparticles

## Abstract

Adoptive cellular therapy (ACT) is a potent strategy to boost the immune response against cancer. ACT is effective against blood cancers but faces challenges in treating solid tumors. A critical step for the success of ACT immunotherapy is to achieve efficient trafficking and persistence of T cells to solid tumors. Non-invasive tracking of the accumulation of adoptively transferred T cells to tumors would greatly accelerate development of more effective ACT strategies. We demonstrate the use of magnetic particle imaging (MPI) to non-invasively track ACT T cells *in vivo* in a mouse model of brain cancer. Magnetic labeling did not impair primary tumor-specific T cells *in vitro,* and MPI allowed the detection of labeled T cells in the brain after intravenous or intracerebroventricular administration. These results support the use of MPI to track adoptively transferred T cells and accelerate the development of ACT treatments for brain tumors and other cancers.

## Introduction

Harnessing the immune system has immense potential for cancer treatment. Several strategies have been explored to boost the immune response against cancer, including cancer vaccines, oncolytic viruses, immune checkpoint blockade, and adoptive cellular therapy (ACT). The last two strategies have shown unprecedented clinical responses and first-in-kind approvals in recent years 1. Adoptive T cell therapy following lymphodepletive conditioning regimens has emerged as one of the most effective treatment strategies against advanced malignant melanoma, with remarkable objective clinical responses in greater than 70% of patients with refractory metastatic disease. This has included a greater than 40% response rate in patients with brain metastases, demonstrating that the central nervous system (CNS) is not refractory to effective treatment with systemically administered T cells 2. Importantly, immunologic surrogate endpoints that correlate with treatment outcome have been identified in these patients, with clinical responses being dependent on the localization of transferred T cells to sites of invasive tumor growth and the persistence of tumor-specific effector memory T cells at high precursor frequency in the blood 3-5. Despite ACT being successful against melanomas and blood cancers, such as leukemia and lymphoma, this therapy has faced challenges in treating solid tumors and invasive CNS malignancies, such as malignant gliomas. One contributing factor lies in the poor trafficking and persistence of T cells in solid tumors following systemic delivery. Therefore, technologies that enable non-invasive and quantitative tracking of adoptively transferred T cells would have a tremendous impact on accelerating the development of new and effective ACT strategies.

Biomedical imaging has been used to track cell therapies, commonly through luminescence/fluorescence imaging, magnetic resonance imaging (MR), positron emission tomography (PET), or single-photon emission computed tomography (SPECT). Cell tracking using these techniques generally follows two approaches: (i) genetic modification of cells to express a marker that enables optical visualization (IVIS®) or nuclear tomographic imaging (PET/SPECT) 6 and (ii) labeling of cells using agents that provide contrast (MR) 7 or generate a signal suitable for imaging (IVIS®, PET/SPECT) 8. However, these commonly used imaging technologies present several disadvantages, such as low cell detection sensitivity, limited tissue penetration depth, low resolution, and artifacts that preclude the precise tracking of cells.

Magnetic particle imaging (MPI) is an emergent biomedical imaging technology that enables the non-invasive visualization of the distribution of biocompatible superparamagnetic iron oxide (SPIO) tracers 9, 10. MPI was first reported in 2005 9 and is rapidly progressing towards clinical translation 11. There is no tissue background with MPI, and signal intensity is proportional to the SPIO mass 12. The signal in MPI does not suffer from the artifacts arising from hypointense SPIO signals observed with MR. Further, the signal in MPI is not attenuated by tissue and has no practical tissue penetration limitations. MPI does not use ionizing radiation, and SPIO tracers have a long shelf life *ex vivo*, while they break down *in vivo*, with iron being incorporated into hemoglobin and ferritin 13, 14. Images obtained from MPI can be co-registered with other modalities, like computed tomography (CT) and MR. Proof-of-principle studies have explored the application of MPI for blood pool imaging 15, 16, lung perfusion 17, 18, bleed detection 19, 20, and tracking of nanoparticle accumulation in cancer tumors 21, 22. To date, cell-tracking studies using MPI have focused almost exclusively on stem cells 10, 23-25.

Here, we evaluate the potential of MPI to track ACT by labeling T cells *ex vivo* with the commercially available magnetic resonance contrast agent ferucarbotran. Ferucarbotran is manufactured by Meito Sangyo (Japan) under the same conditions as Resovist and is marketed in the US as VivoTrax™ by Magnetic Insight for use in MPI. While ferucarbotran is not optimized for MPI, it has been widely adopted in MPI studies due to its commercial availability. We assess the effects of ferucarbotran labeling on the viability, phenotype, and effector function of tumor-specific primary T cells. The intracellular localization of ferucarbotran is evaluated using complementary microscopy techniques. T cell uptake of ferucarbotran is quantified using MPI and validated against a well-established iron quantification assay. The linearity between MPI signal and labeled T cell count is demonstrated. *In vivo* MPI shows ferucarbotran signal accumulation in the tumor-bearing brain following ACT, and the *ex vivo* co-localization of this MPI signal and T cell reporter fluorescence suggests that MPI can detect the accumulation of live ferucarbotran-labeled T cells in the brain of tumor-bearing mice following ACT. For mice receiving T cells through different routes of injection, *e.g*., intravenous or intracerebroventricular, *in vivo* MPI permits the tracking of their localization over time. Therefore, these studies demonstrate that ferucarbotran-labeled T cells can be imaged non-invasively using MPI.

## Results

### T cell viability, cytotoxic phenotype, and effector function are unaffected by labeling with ferucarbotran

**Figure 1:**
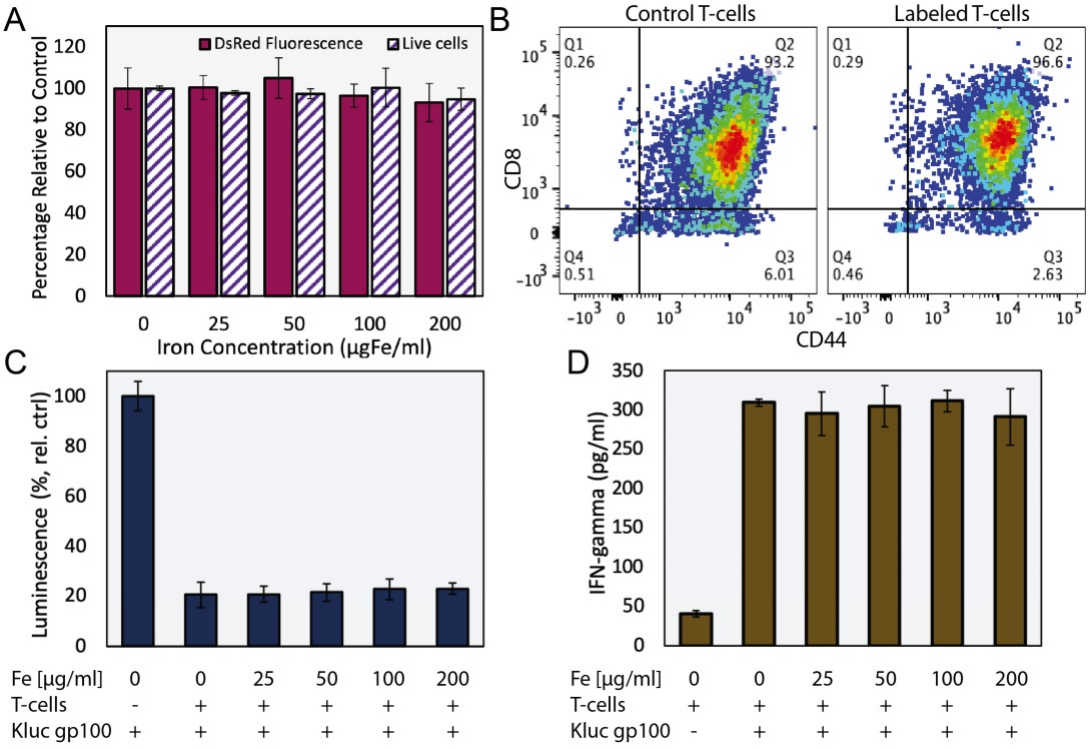
** Ferucarbotran labeling does not impair T cells.** A) Ferucarbotran-labeled T cell relative to control, cell viability as determined by trypan blue exclusion assay and DsRed fluorescence measurements at different iron labeling conditions. B) Phenotyping of control and labeled (100 µgFe/ml) T cells by flow cytometry. C) Relative viability of Kluc-gp100 cells after co-culture with ferucarbotran labeled T cells, as determined from Kluc-derived luminescence relative to control (no T cells in co-culture). D) IFN-γ release of T cells in co-culture with KLuc-gp100 tumor cells, as determined by ELISA.

We evaluated the effects of ferucarbotran labeling on the viability and phenotype of pmel DsRed T cells incubated with various concentrations of ferucarbotran (0 to 200 µgFe/ml) for 24 h. Cell viability and DsRed fluorescence were not affected by labeling with ferucarbotran, as determined with trypan blue exclusion assay and fluorescence readings, respectively (**Fig. 1A**). T cell phenotype was evaluated using flow cytometry (**Fig. 1B**). Here, CD8+/CD44+ markers were used to distinguish between activated and naïve T cells and determine if differences in phenotype were seen after labeling with ferucarbotran. The expression of CD8/CD44 on the unlabeled control (n=4), and ferucarbotran-labeled T cells (n=4) was more than 97% of the CD3+ lymphocyte population. The expression of CD27 in both unlabeled and ferucarbotran-labeled T cells is less than 0.2%, suggesting that T cells are effector and not memory T cells. These results show that ferucarbotran labeling does not affect viability or phenotype of pmel DsRed T cells at the studied iron concentrations.

Cytotoxic activity of ferucarbotran-labeled pmel DsRed T cells against Kluc-gp100 glioblastoma cells was assessed by co-culture of T cells with tumor cells (5:1 ratio) for 24 h. Kluc-gp100 luciferase expression or IFN-γ were measured in separate experiments. A reduction in luciferase expression by the Kluc-gp100 cells in co-culture with T cells would result from a reduction in tumor cell viability due to the cytotoxic activity of the T cells. Results show that labeling T cells using ferucarbotran does not impair their ability to kill Kluc-gp100 cells (**Fig. 1C**), or their ability to produce IFN-γ (**Fig. 1D**), for all the ferucarbotran labeling conditions used. No statistically significant differences were observed at any given iron concentration in co-cultured groups as compared with the co-culture control (0 µgFe/ml) using one-way ANOVA. Statistically significant differences were observed from co-culture conditions as compared to the negative control (no co-culture) for both luminescence (p value < 0.0001) and IFN-γ assay (p value < 0.0001).

### Ferucarbotran associates with the T cell membrane and is internalized, leading to measurable MPI signal

**Figure 2:**
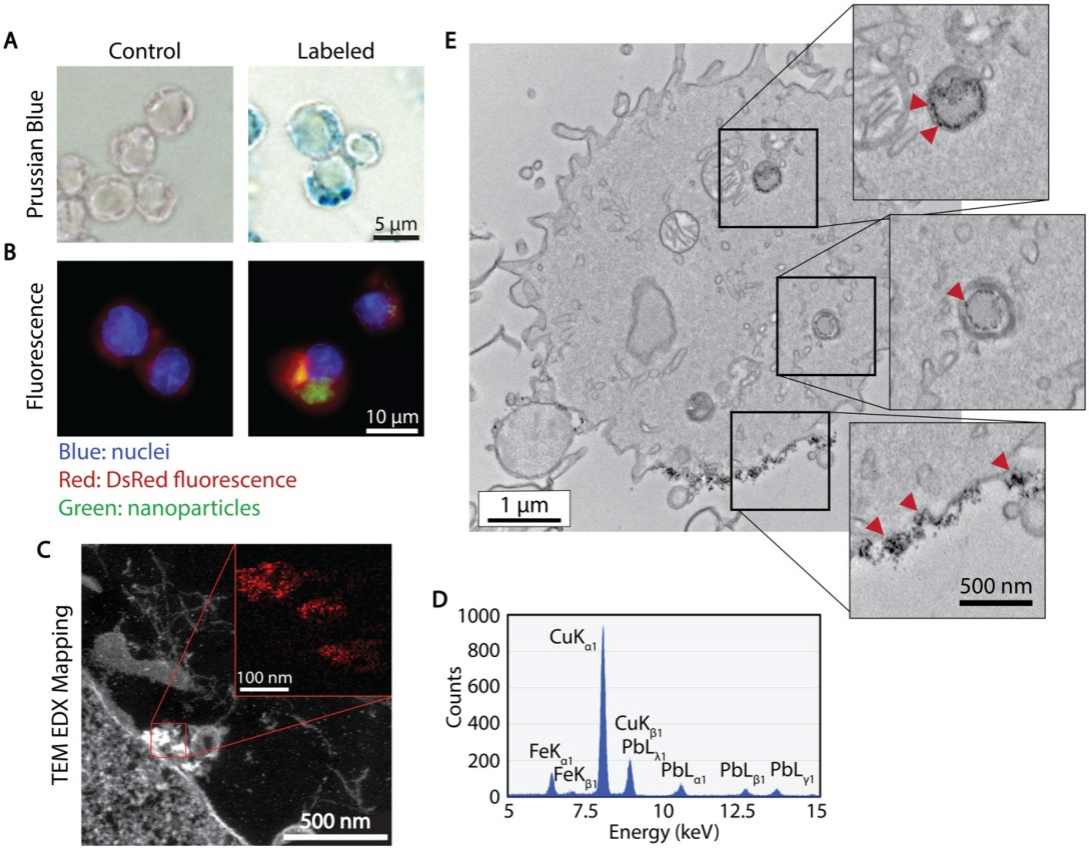
** Cellular localization of ferucarbotran tracers in T cells.** A) Prussian blue stain for iron oxide. B) Immunofluorescent labeling of ferucarbotran dextran coating (blue- nuclei, red- DsRed fluorescence, green- dextran). C) Z-contrast and energy dispersive X-ray (EDX) spectroscopy show ferucarbotran tracers (bright signal and red in inset) in T cells. D) EDX spectrum shows Fe from ferucarbotran, Cu from the grid, and Pb from stain. E) Ferucarbotran tracers are seen in intracellular vesicles (top two insets) and associated with the cell membrane (bottom inset). Cell section stained with osmium only (no Pb) to improve ferucarbotran contrast.

The association and uptake of ferucarbotran nanoparticles by T cells were evaluated using complementary microscopy techniques. Prussian blue was used to detect iron deposits in cells using optical microscopy. **Fig. 2A** shows iron deposits detected by Prussian blue staining in the ferucarbotran labeled T cells, whereas staining was absent in control cells. The uptake of ferucarbotran was further evaluated using fluorescence microscopy and an anti-dextran FITC antibody specific for the carboxydextran coating of the nanoparticles (**Fig. 2B**). Particles within T cells are shown as green in fluorescence microscopy. Since the T cells endogenously express reporter DsRed (red) fluorescence, live-cell imaging was performed, and nuclei were stained using Hoechst 33342 (blue). Immunostaining for dextran was observed in the ferucarbotran-labeled group only. Together, Prussian blue and fluorescence microscopy demonstrate the labeling of T cells with ferucarbotran.

To further evaluate the uptake and intracellular localization of ferucarbotran within the T cells, transmission electron microscopy (TEM) and energy dispersive x-ray (EDX) spectroscopy were used. **Fig. 2C** shows z-contrast TEM and elemental mapping of Fe distribution (inset, Fe in red). The EDX spectrum (**Fig. 2D**) confirms the presence of iron, copper from the TEM grid, and lead staining for enhanced cell organelles contrast. Since the use of lead staining makes visualization of ferucarbotran inside T cells challenging, ferucarbotran internalization was also confirmed in sections stained only with osmium, to improve the contrast between ferucarbotran and cellular components (**Fig. 2E**). Ferucarbotran is observed associated with the cell membrane and also in intracellular vesicle-like structures.

### MPI signal quantifies ferucarbotran T cell labeling and is linear with the number of labeled T cells

**Figure 3:**
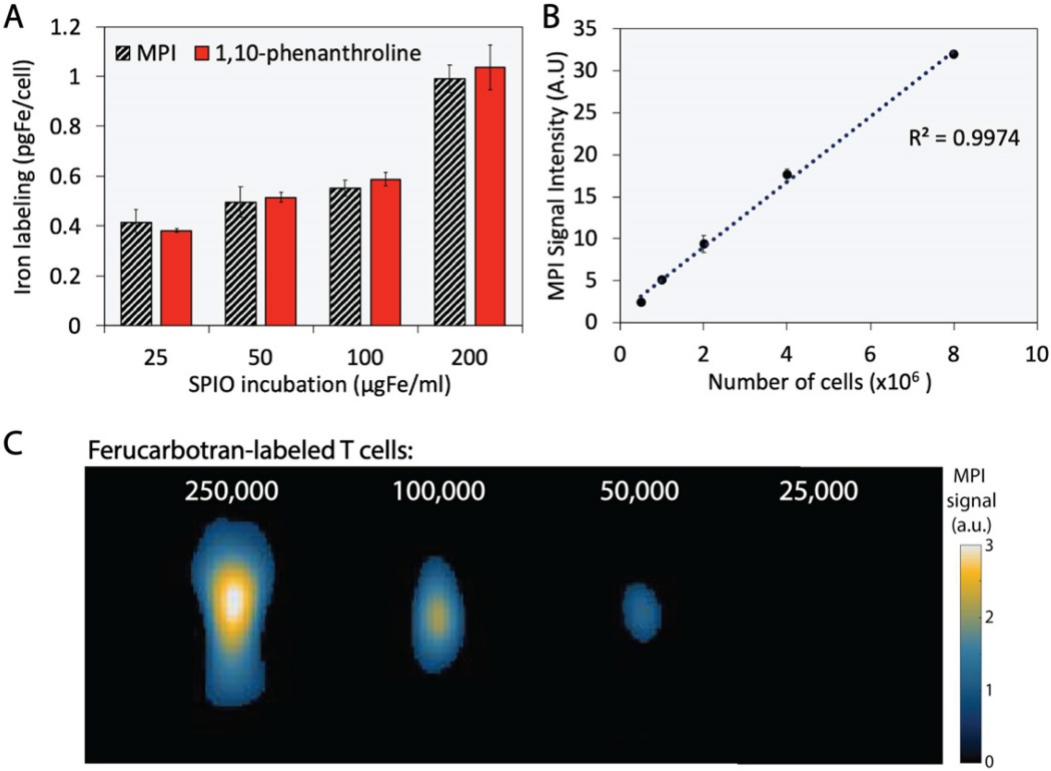
** Ferucarbotran-labeled T cells can be quantified using MPI.** A) Iron content per cell, quantified using 1,10-phenanthroline assay and MPI. B) Linear correlation between MPI signal intensity and number of ferucarbotran-labeled T cells. Error bars correspond to standard deviation (n=3). Some error bars are smaller than the markers. C) 2D maximum intensity projection scans for samples containing decreasing numbers of ferucarbotran-labeled T cells. Note: For MPI 2D high sensitivity scans 1 a.u. is equivalent to 1.29 ngFe/mm^2^ when ferucarbotran is used as tracer.

The extent of T cell labeling with ferucarbotran was quantified using the 1,10-phenanthroline colorimetric assay and MPI. Ferucarbotran uptake was determined using MPI by comparing the cell sample MPI signal with the signal obtained from a fiducial with free ferucarbotran of known iron mass. We found that ferucarbotran uptake ranges from ~0.4 to ~1 pgFe/cell, depending on the ferucarbotran concentration used during the 24 h incubation period. Of significant interest, the excellent agreement between ferucarbotran uptake determined using MPI or the 1,10-phenanthroline assay suggests that MPI signal per iron mass is not affected after T cell uptake (**Fig. 3A**). Linearity between MPI signal and number of ferucarbotran-labeled T cells was evaluated. Ferucarbotran mass in the sample was quantified through image analysis using a fiducial with known mass. An excellent linear correlation between MPI signal intensity and the number of ferucarbotran-labeled T cells was observed (**Fig. 3B**). 2D-MPI scans of samples with different numbers of ferucarbotran-labeled T cells demonstrated that 5×10^4^ cells could be detected in the MPI (**Fig. 3C**). The limit of detection determined for ferucarbotran in our MPI system was approximately 38 ngFe (**Fig. S1** and **Table S1**).

### MPI allows the *in vivo* detection and tracking of adoptively transferred ferucarbotran-labeled T cells

**Fig 4:**
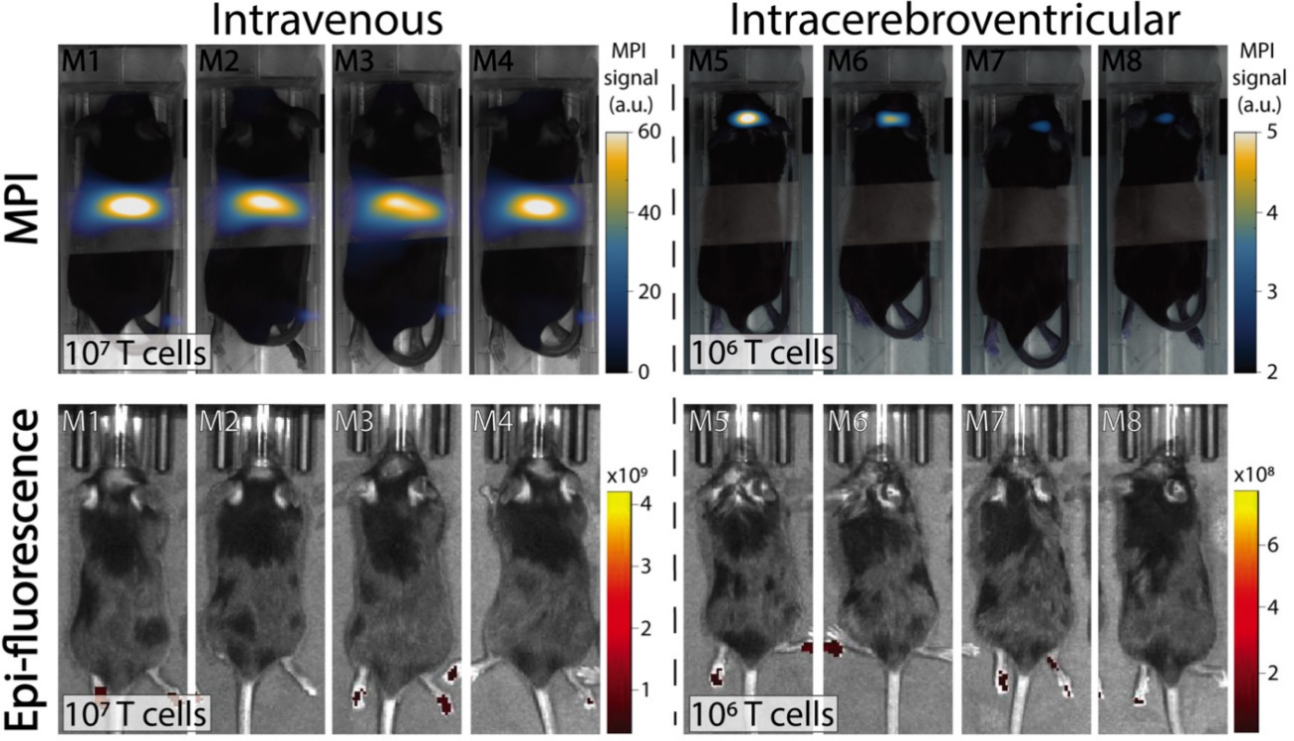
** Comparison of IVIS® and MPI tracking of ferucarbotran-labeled T cells in mice bearing intracranial glioma tumors.** Mice received T cells intravenously via the tail vein (10^7^ T cells in 100 µL PBS, n=4) or intracerebroventricularly (10^6^ T cells in 5 µL, n=4) using a stereotactic stage, and were imaged using IVIS® and MPI 24 h post ACT injection. IVIS® signal in paws represents tissue autofluorescence noise. Results clearly show that ferucarbotran-labeled pmel DsRed T cells can be tracked *in vivo* using MPI, whereas IVIS® fluorescence fails to capture ACT T cell biodistribution. Note: mice are labeled with M# to identify the same mouse in each imaging modality; the white band in MPI images is adhesive tape used to keep the mice in the same position while imaging. Note: For MPI 2D high sensitivity scans 1 a.u. is equivalent to 1.29 ngFe/mm^2^ when ferucarbotran is used as tracer. Units of epifluorescence are in radiant efficiency [(p/sec/cm^2^/sr)/(µW/cm^2^)].

*In vivo* experiments were performed in C57BL/6 mice bearing intracranial KLuc-gp100 tumors. Ten days after cancer cell implantation, ACT was performed using ferucarbotran-labeled T cells delivered via the tail vein (10^7^ cells in 100 µL) or intracerebroventricularly (10^6^ cells in 5 µL). After injection, T cell biodistribution was monitored with IVIS® using T cell endogenous DsRed reporter fluorescence, and MPI to detect the ferucarbotran-labeled T cells (**Fig. 4**). Using MPI, T cell accumulation after intravenous administration was initially detected in the lungs and liver. In contrast, *in vivo* fluorescence imaging failed to detect T cells in those organs, even immediately after administration. For the intracerebroventricular injection of ferucarbotran-labeled DsRed T cells, IVIS® fluorescence imaging failed to detect T cells despite these cells being concentrated at a point source, where they were delivered in the brain. However, a clear MPI signal was observed at the site of injection.

### Multimodal MPI/CT imaging allows the non-invasive evaluation of T cell localization in the brain after intraventricular administration

**Figure 5:**
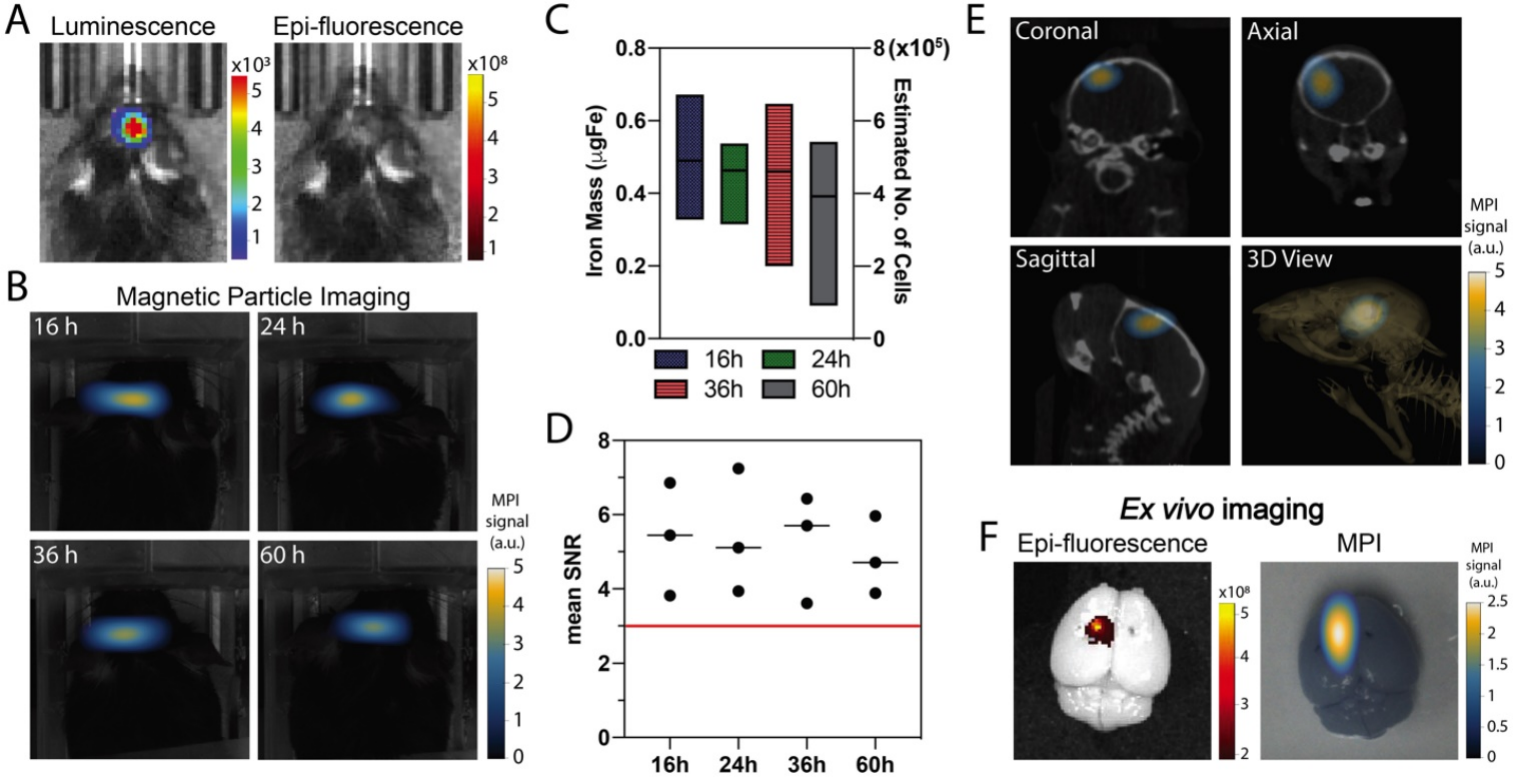
***In vivo* and *ex vivo* imaging after intracerebroventricular administration of ferucarbotran-labeled T cells.** A) *In vivo* imaging shows the presence of an intracranial glioblastoma kluc-gp100 tumor (luminescence). After intracerebroventricular injection of labeled T cells, *in vivo* DsRed fluorescence signal is not visible (36 h) due to tissue attenuation. B) Time series of *in vivo* MPI shows a consistent MPI signal at the site of injection (n=3). C) Box plot with iron mass in brain tumor at different time points estimated based on MPI RoI analysis and corresponding estimated number of cells based on labeling at 1 pgFe/cell. D) Mean signal-to-noise (SNR) ratio shows that MPI signal is above the noise level (red line). E) MPI co-registered with CT scans, with different anatomical plane cross-sections showing the intracranial localization of ferucarbotran-labeled T cells. F) *Ex vivo* fluorescence signal colocalized with MPI signal. Note: For MPI 2D high sensitivity scans 1 a.u. is equivalent to 1.29 ngFe/mm^2^ when ferucarbotran is used as tracer. Units of luminescence are in radiance [(p/sec/cm^2^/sr)]. Units of epifluorescence are in radiant efficiency [(p/sec/cm^2^/sr)/(µW/cm^2^)].

Co-registration of MPI and computed tomography (CT) enabled evaluation of the intracranial persistence of ferucarbotran-labeled T cells over time following intracerebroventricular injection. *In vivo* luminescence confirmed the presence of Kluc-gp100 brain tumor (**Fig. 5A**). Ferucarbotran-labeled DsRed T cells (10^6^) were injected in the lateral ventricles, and epifluorescence and MPI imaging were performed at different time points after injection. *In vivo* DsRed epifluorescence signal was not detected at any time point (**Fig. 5A** shows epifluorescence at 36 h). However, MPI signal was detected and monitored *in vivo* at different time points (**Fig. 5B**), showing an apparent decrease over time in the number of T cells estimated from the MPI signal (**Fig. 5C**). However, at the studied time points, these changes are not statistically significant (p value of 0.9462). The injected labeled T cells have 1 pgFe/cell and around 10^6^ cells were injected in each mouse, corresponding to ~1 ugFe delivered to the brain. MPI quantification suggests a lower amount (~0.4-0.6 ugFe) was actually delivered. **Fig. 5D** shows that mean signal-to-noise ratio for each of the measurements is consistently above 3. *In vivo* 3D MPI scans were acquired, followed by CT scans. This allows co-registration to demonstrate location and orientation of the MPI signal/labeled T cells in the brain using 3D anatomical references from CT-scans (**Fig. 5E**). *Ex vivo* imaging was also performed, which enabled epifluorescence visualization of DsRed T cells in the brain because of reduced signal attenuation due to the absence of hair, skin, and bone. The MPI signal from ferucarbotran-labeled T cells was co-localized with the *ex vivo* DsRed fluorescence signal, suggesting that both signals arise from T cells in the brain (**Fig. 5F**).

### *In vivo* MPI and *ex vivo* MPI and fluorescence imaging suggest that viable ferucarbotran-labeled T cells are detectable in brain tumors using MPI following systemic injection

**Figure 6:**
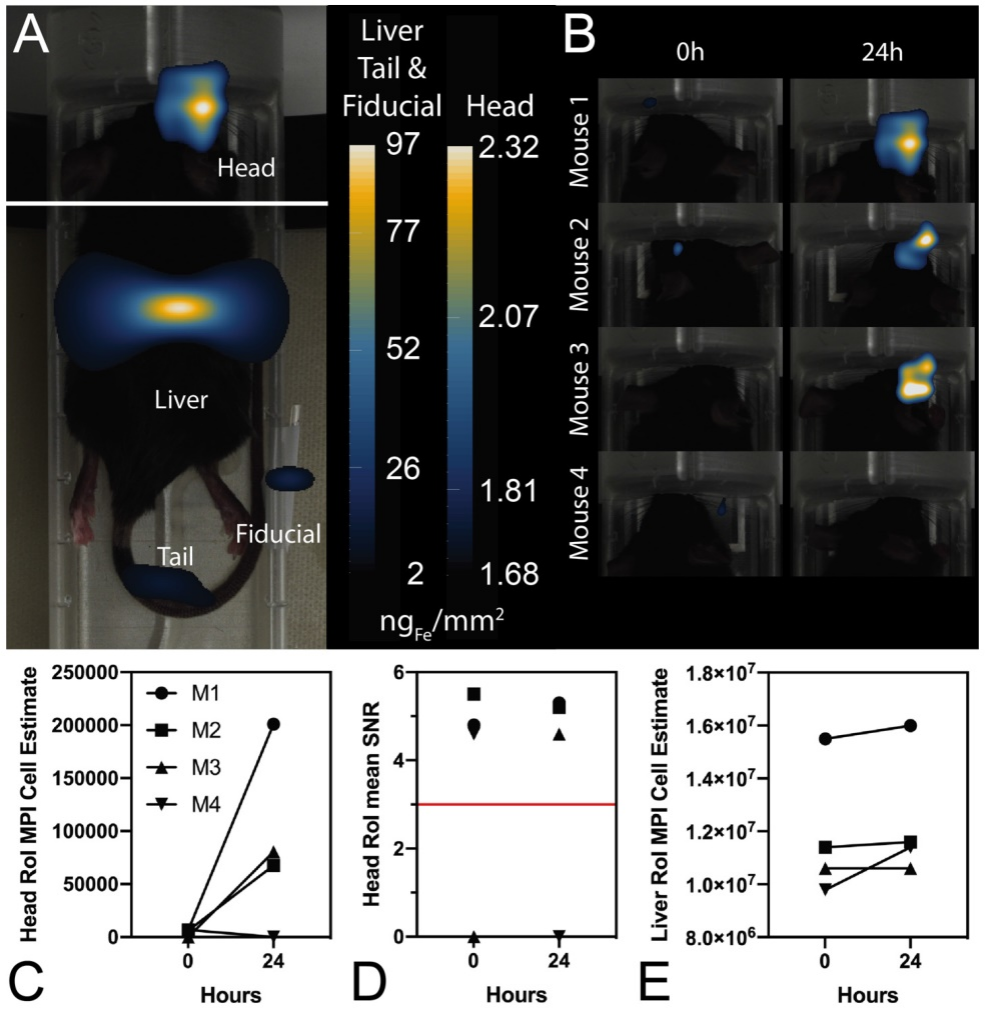
** Ferucarotran-labeled tumor-specific T cells can be detected *in vivo* using MPI.** A) MPI shows *in vivo* accumulation of ferucarbotran-labeled T cells in the brain and liver 24 h after ACT administration (n=4). B) Increase in MPI signal after 24 h suggest that T cells accumulate in the tumor. C) MPI signal quantifies the number of ferucarbotran-labeled T cells in the head. D) Mean signal-to-noise ratio (mSNR) in the head region of interest (RoI). E) MPI signal quantifies the number of ferucarbotran-labeled T cells in the liver.

**Fig 7:**
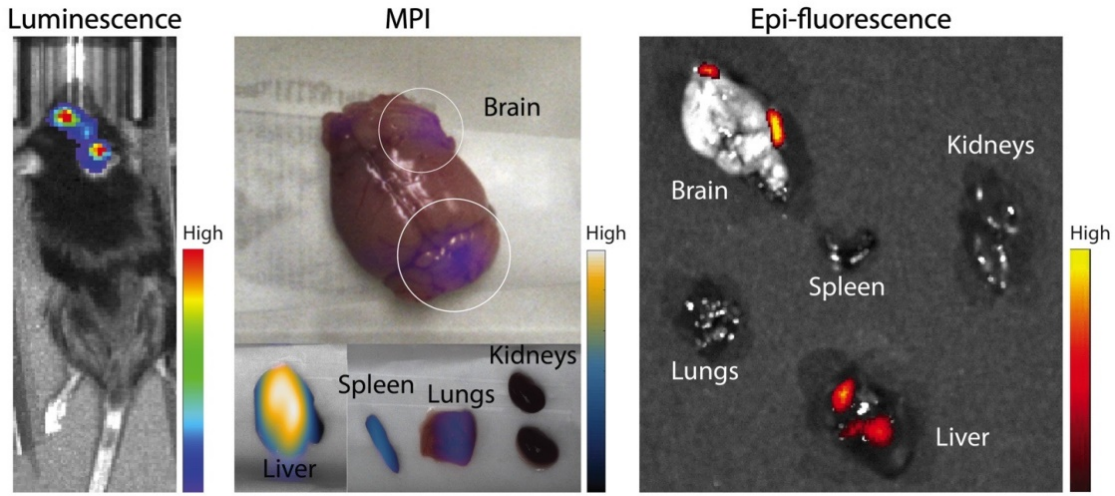
** Ferucarbotran-labeled tumor-specific T cells can be detected *ex vivo* using MPI.** *Ex vivo* MPI and fluorescence imaging show the accumulation of T cells in two areas of the brain and different organs.

**Fig 8:**
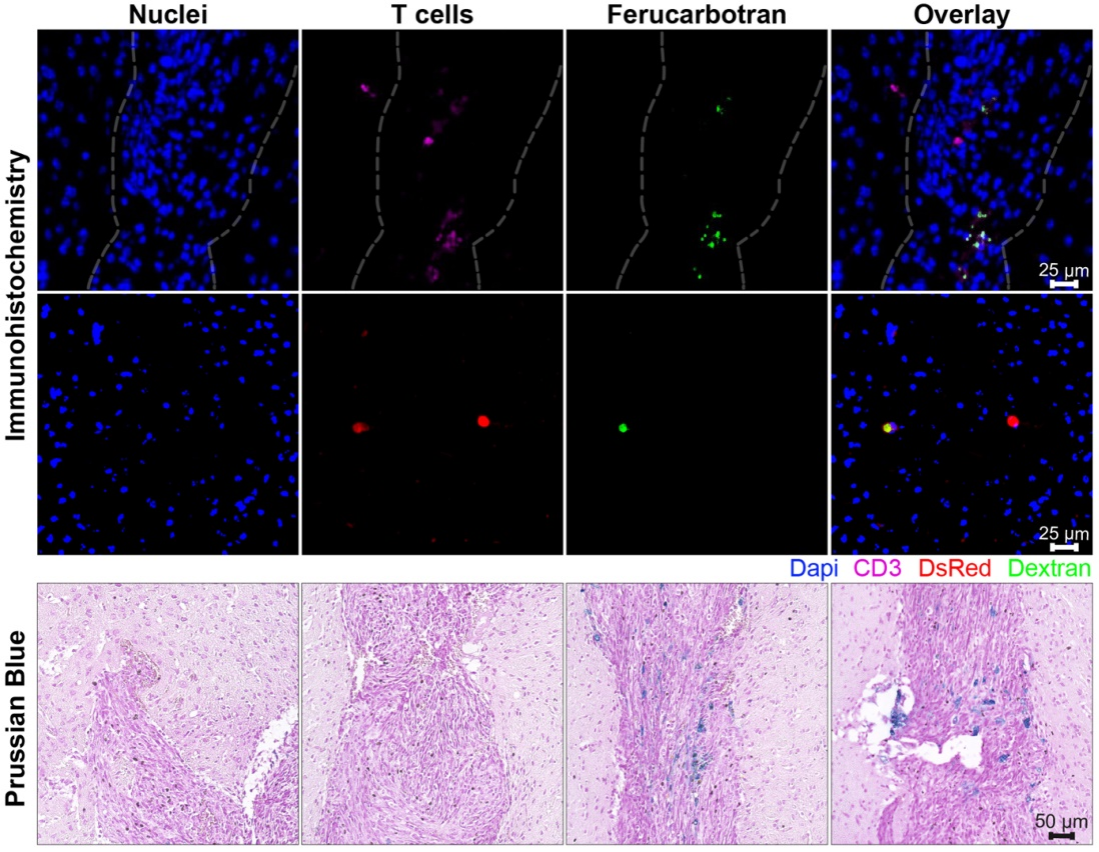
Ferucarbotran-labeled T cells can be visualized in histological sections of the brain after intravenous administration. Top panel shows immunohistochemistry of brain tumor histological sections that were stained with DAPI for nuclei (blue), anti-CD3 for T cells (magenta), anti-DsRed (RFP, red) for T cells, and anti-dextran FITC (green) for ferucarbotran dextran coating. Tumor area with dense nuclei region is demarcated with dashed line. Bottom panel shows Prussian Blue staining for iron oxide with four regions (A-D) of the brain tumor are shown at higher magnification.

In further *in vivo* experiments, ferucarbotran-labeled T cells were injected intravenously (10^7^ cells in 100 µL PBS) 25 days after cancer cell implantation in the brain. After injection, T cell biodistribution was monitored using DsRed fluorescence in IVIS® and with MPI. Using MPI, T cell accumulation was initially detected in the liver (**Fig 6A**). An increase in MPI signal was observed 24 h after injection in the brain of 3 out of 4 tumor-bearing animals (**Fig. 6B**) and the number of cells in the brain was estimated (**Fig. 6C**) based on a fiducial calibration curve and the assumption that the cells contain 1 pgFe/cell, as determined *ex vivo*. **Fig. 6D** shows the mean signal-to-noise ratio (mSNR) for the MPI signal in the head for all animals and all time points. Small regions were observed with mSNR greater than 3 in three of the four animals at 0 h, however, these regions were small and total signal was correspondingly negligible. In contrast, mSNR was high for relatively large regions in three of the four animals at 24 h and the corresponding estimated number of cells in the RoI were above the *ex vivo* limit of detection of 50,000 cells. **Fig. 6E** shows the estimated number of T cells that accumulated in the liver, which agrees well with the dose of 10^7^ cells. In contrast, fluorescence imaging failed to detect T cells *in vivo* for all animals and time points. Organs were harvested 24 h after T cell tail vein injection and evaluated using MPI and IVIS®. *Ex vivo* MPI showed the accumulation of ferucarbotran-labeled T cells in the liver, spleen, lungs, and brain, whereas IVIS® imaging of DsRed fluorescence detected T cells in the brain and liver (**Fig. 7**). The presence of ferucarbotran-labeled T cells in the brain was also demonstrated through histological sections. **Fig. 8** (top panel) shows immunohistochemistry of the brain that was stained with anti-CD3 (magenta) and anti-DsRed (red) for T cells, anti-dextran FITC (green) for the ferucarbotran dextran coating, and DAPI (blue) to identify the dense nuclei of the brain tumor. Co-localization of CD3 (magenta) and dextran (green), and DsRed (red) and dextran (green) suggests that ferucarbotran-labeled T cells were able to reach the brain tumor. Also, in **Fig. 8** (bottom panel) Prussian Blue staining can be observed in the tumor region of the brain, demonstrating the presence of iron oxide nanoparticles.

## Discussion

To examine the effects of ferucarbotran labeling on T cell viability, phenotype, and function, cytotoxic gp100/pmel-specific DsRed T cells were harvested from the spleen of transgenic mice, activated, expanded *ex vivo,* and labeled with ferucarbotran. These transgenic T cells are specific for an epitope of the melanoma tumor antigen gp100 expressed by murine B16 melanomas and GL261 gliomas 26, 27. The clinically relevant intracranial murine glioma cell line KR158B was modified to express luciferase and gp100 (KR158B-Luc-gp100; Kluc-gp100), while retaining the high-grade glioma features of the parental cell line. After intracranial implantation, the KR158B-Luc-gp100 cell line forms islands of invasive tumor infiltrates that closely resemble those observed in human gliomas and is refractory to radiation and chemotherapy 28. The gp100 antigen expressed by this Kluc-gp100 glioblastoma cell line is specifically recognized by the pmel-specific DsRed T cells we used in our cell tracking studies.

Ferucarbotran tracer was used to label murine T cells for *in vivo* tracking following transfer in the immunocompetent murine Kluc-gp100 glioblastoma model. Ferucarbotran is a mixture of Fe_2_O_3_ and γ-Fe_3_O_4_ nanoparticles with a core size of ~5 nm, in a carboxydextran matrix with a hydrodynamic diameter of 40-60 nm and negative ζ- potential 29, 30. These particles were developed as a magnetic resonance contrast agent 29, 31, and are not optimized for MPI. However, ferucarbotran is available commercially, has acceptable MPI tracer properties, and serves as a platform for proof-of-principle studies and comparisons across groups.

In the field of cell tracking technologies, it is important to evaluate the effects of the imaging agent on the tracked cells. For ACT T cell tracking, it is imperative that the tracer and labeling method do not affect the cellular processes involved in the tumor specificity and effector function of immune cytotoxic T cells. Here, we demonstrated that labeling primary T cells with ferucarbotran nanoparticles does not affect, under the studied conditions, T cell viability, phenotype, or cytotoxic effector function. This was shown through the analysis of phenotypic markers using flow cytometry. For phenotyping, it was previously demonstrated that activation using Concanavalin A favors T cell expansion towards CD8 and CD44 expression, corresponding to effector/effector memory T cells 32. We showed that the expression of CD8/CD44 in the control and labeled T cells remains the same, suggesting labeling with ferucarbotran does not affect the CD8 co-receptor, which is a marker for cytotoxic T cell populations and plays a role in T cell signaling and antigen interactions. Similarly, CD44, which is a prominent activation marker, is used to distinguish memory and effector T cells from their naïve counterparts (**Fig. 1**). CD27 expression on CD8+ T cells is lost after repetitive antigenic stimulation 33, and the low expression of CD27 on control and labeled T cells suggest that these T cells are effector and not memory T cells. Furthermore, T cell effector function was evaluated by quantifying the release of immunostimulatory cytokine interferon-gamma (IFN- γ), which is involved in the anti-tumor cytotoxic T cell response 32, and by evaluating cancer cell killing by T cells in co-cultures. A reduction in the luminescence of Kluc-gp100 cells in co-cultures would result from their killing due to the anti-tumor activity of CD8 T cells, which is enhanced by the release of IFN-γ 32. Our data show that ferucarbotran labeling does not impair the ability of T cells to produce IFN-γ and kill Kluc-gp100 glioma cells, in all of the ferucarbotran labeling conditions used.

The association and uptake of ferucarbotran-coated nanoparticles by T cells were evaluated using complementary microscopy techniques. Here, we demonstrated that ferucarbotran nanoparticles, which have a carboxydextran coating, are taken up by T cells in vesicle-like structures while also remaining in association with the T cell membrane (**Fig. 2**). Other studies on cell labeling with magnetic nanoparticles have used dextran-coated particles and have reported insufficient cellular uptake, claiming steric repulsion between particles and cell membrane 34. While this will certainly depend on the cell type and its ability to phagocytose particulates, very little is known about nanoparticle uptake by primary T cells.

We found that ferucarbotran uptake is up to 1 pgFe/cell at incubation conditions of 200 µgFe/ml for 24 h. We quantified iron labeling by using the 1,10-phenanthroline colorimetric assay, which involves sample digestion in a two-day process. The working principle of this assay is to form iron (II)- ortho-phenanthroline complexes following iron (III) reduction. Absorbance is then measured at 508 nm. This was compared to iron oxide mass quantification using MPI, which can be applied to quantify ferucarbotran mass in live or dead cells, in a process that takes ~15 min for sample preparation, image acquisition, and analysis. The excellent agreement between both quantification methods suggests that MPI signal per iron mass is not affected after T cell uptake (**Fig. 3**). This result is in contrast to a recent report by Suzuka *et al*. suggesting that ferucarbotran MPI signal is affected after uptake into macrophages or colon cancer cells 35. We note that the discrepancy between our observations and those of Suzuka *et al*. may be due to differences in excitation frequency between their prototype scanner (0.4 kHz) and the Momentum^TM^ scanner used here (45 kHz). Ferucarbotran tracer MPI response may be more susceptible to cell uptake at the lower excitation frequencies used in the study by Suzuka *et al*., in comparison to the higher excitation frequencies used in the present study and previous cell-tracking studies 23, 24. Additionally, there might be differences in uptake and cellular localization patterns between the T cells used here and the macrophages or colon cancer cells used by Suzuka *et al*., which could contribute to this difference in observations.

We note that cell detection sensitivity in MPI is determined by the intrinsic signal per iron mass of the tracer used and the extent of cell labeling with the tracer. In the present study, the cell detection sensitivity was approximately 50,000 cells. Much lower cell detection sensitivity of approximately 200 cells has been reported for stem cells using MPI 24. However, in that report, the stem cells had taken up ~27 pgFe/cell. Furthermore, that study used a prototype MPI scanner, rather than the commercially available MPI scanner used here. Other reports show ~2.43 pgFe/cell for ferumoxytol-labeled mesenchymal stem cells 36. While the T cell uptake of ferucarbotran was relatively low at ~1 pgFe/cell, it is important to consider that not all cell types are phagocytic or prone to uptake particulates from their environment. Also, differences in labeling strategies, such as incubation period, use of transfection agents, particle concentration, among others are important factors that will affect labeling and sensitivity 37. In addition, lymphocytes are relatively small in size, with an approximate cell volume of 125 µm^3^ and with most of its cytoplasmic space occupied by the nuclei, as compared with the volume of a typical cell (~4000 µm^3^) 38. We expect that better T cell sensitivity can be obtained by increasing T cell labeling with the MPI tracer. Additionally, better cell detection sensitivity can be obtained through the use of tracers with higher signal per iron mass, by tuning imaging parameters, and MPI hardware 39. For example, the tracer LS-017, which was optimized for MPI, was reported to have 6 times higher signal intensity than ferucarbotran 40, with further improvements in MPI tracer signal being possible in this field.

As with other cell tracking techniques that involve the direct labeling of cells, two main challenges of this approach are the dilution of the tracer among the cell progeny and the release of nanoparticles after apoptosis. Furthermore, cell tracking using MPI cannot at present distinguish between live and dead cells, as dead cells might still retain nanoparticles. However, this limitation can be overcome by combining cell labeling using MPI tracers with genetically engineered T cells containing reporter genes to enable multi-modal imaging of their distribution (using MPI) and viability (using luminescence/fluorescence), as shown here. *In vivo* experiments were performed using C57BL/6 mice bearing intracranial Kluc-gp100 tumors. Ferucarbotran-labeled pmel DsRed T cells were injected either intravenously through the tail vein or intracerebroventricular (local delivery). IVIS® and MPI cell tracking were performed using the DsRed gene reporter and ferucarbotran-labeling, respectively (**Fig. 4**). For all the *in vivo* imaging, IVIS® epifluorescence failed to detect DsRed signal, whereas MPI was able to detect T cell biodistribution *in vivo*.

Because signal in MPI only arises from the superparamagnetic iron oxide nanoparticles, co-registration with another imaging modality is needed for anatomical reference, in a situation that is similar to that with nuclear imaging. In the present study, we used optical images and CT-scans as a means to co-register MPI using fiducials as references. MPI allowed monitoring of intracerebroventricular adoptively transferred T cells, a clinically feasible administration route that resulted in positive clinical response 41, and is currently part of Phase 1 trials 42, 43. Here, we show a decrease in iron mass over 60 h post-administration, which can be related to *in vivo* tracer dilution in the cell progeny, particle exocytosis, or particle degradation *in vivo* (**Fig. 5C**). In contrast, DsRed epifluorescence was not detected *in vivo* due to signal attenuation, despite the fact the DsRed labeled T cells were locally delivered in high numbers (10^6^ cells). MPI/CT co-registration enabled evaluation of the intracranial localization of ferucarbotran labeled T cells (**Fig. 5E**). *Ex vivo* imaging enabled epifluorescence visualization of DsRed T cells in the brain because of reduced signal attenuation due to the absence of hair, skin, and bone. The MPI signal from ferucarbotran-labeled T cells was co-localized with the *ex vivo* DsRed fluorescence signal, suggesting that both signals arise from T cells in the brain (**Fig. 5F**).

For experiments with intravenous ACT administration, ferucarbotran-labeled T cells were initially detected in the liver and an increase in MPI signal was observed 24 h after injection in the brain of 3 out of 4 animals (**Fig. 6**). These observations suggest that tumor-specific labeled T cells accumulate in the brain tumor *in vivo* and the signal can be detected using MPI. Furthermore, the number of cells in the tumor were estimated using MPI (**Fig. 6C**). In the three mice where a signal increase was observed, an estimate of 7×10^4^ to 2×10^5^ cells where quantified at 24h post-ACT, above the limit of detection of 5×10^4^ determined *ex vivo* (**Fig. 3**). Although fluorescence failed to detect *in vivo* signal, DsRed fluorescence was observed in the brain *ex vivo* at 24 h after tail vein injection. The DsRed *ex vivo* signal co-localized with the *ex vivo* MPI signal in the brain, in addition to other major organs such as the liver, spleen, and lungs (**Fig. 7**). *Ex vivo* MPI and fluorescence detection was confirmed through histological sections of the brain (**Fig. 8**), which demonstrate the presence of CD3 and DsRed T cells in the brain tumor, colocalizing with the dextran coating from the ferucarbotran nanoparticles. In addition, Prussian Blue staining also confirmed the presence of iron deposits in brain tissue. These observations suggest that MPI was capable of detecting accumulation of viable ferucarbotran-labeled adoptively transferred T cells in the brain of tumor-bearing mice.

There are several limitations of this study that point towards future work. As a first proof-of-principle study, we elected to label the T cells with the commercially available tracer ferucarbotran. This was expedient due to the ease of availability of the tracer. It is also relevant to the MPI community as ferucarbotran has been used in prior studies, enabling comparisons. However, ferucarbotran was not developed or optimized for MPI. In fact, studies suggest that only a small fraction of the SPIOs in ferucarbotran contribute to its MPI signal 44. Because cell tracking sensitivity depends on tracer signal magnitude, we expect that even better MPI T cell sensitivity will be possible with tracers optimized for MPI. We are currently evaluating other commercially-available tracers and developing our own for this purpose. Furthermore, while our results show that primary T cells can be labeled using ferucarbotran, the extent of labeling is low (~1 pgFe/cell) compared to other cells. Here we expect that improved understanding of nanoparticle-T cell interactions, and engineering of nanoparticle surface coatings and functionality could lead to improved uptake. This, in turn, will lead to improved T cell tracking sensitivity using MPI. Finally, as with other direct labeling techniques, the MPI signal in SPIO labeled T cells is subject to dilution due to cell division and reduction due to tracer exocytosis or degradation. Very little is known about the fate of nanoparticles taken up by T cells, whether *in vitro* or *in vivo*. Improved understanding will help interpret T cell tracking studies using MPI, particularly over long time periods. However, despite these limitations, our results strongly suggest that MPI is an attractive approach to track T cells and could provide a unique insight into the fate of ACT T cell cancer immunotherapies.

## Materials and Methods

### Animals and cell lines

C57BL/6 wild type mice were obtained from Jackson Laboratory. Transgenic pmel specific DsRed C57BL/6 mice were obtained from breeding a DsRed transgenic mouse (B6.Cg-Tg(CAG-DsRed*MST)1Nagy/J) and a pmel transgenic mouse (B6.Cg-Thy1a/Cy Tg(TcraTcrb)8Rest/J) to obtain the DsRed pmel-specific mouse colony. The pmel-specific T cell receptor of these mice recognizes the gp100 antigen expressed by the studied murine glioblastoma cell line, and the DsRed fluorescence allows *ex vivo* identification of transferred T cells. The murine glioblastoma cell line KR158B-Luc was a kind gift from Tyler Jacks 45. The KR158B-Luc cell line was genetically modified to express gp100 (Kluc-gp100) using the lentiviral vector pD2107-CMV-gp100. Kluc-gp100 cells were cultured in Dulbecco's Modified Eagle Media (DMEM) containing 10% fetal bovine serum (FBS). All animals were housed in specific pathogen-free facilities. Experiments were performed according to the University of Florida Institutional Animal Care and Use Committee (IACUC) approved protocols.

### T cell isolation and activation

Spleen was harvested from 4- to 8-week-old pmel DsRed transgenic mice. Cells were cultured in Roswell Park Memorial Institute (RPMI) 1640 (Gibco, Waltham, MA) medium supplemented with 10% FBS, 1% non-essential amino acids, 1% L-glutamine, 1% sodium pyruvate, 0.1% 2-mercaptoethanol, 1% penicillin/streptomycin, and recombinant human IL-2 (50 U/mL, R&D Systems, Minneapolis, MN, USA). Single-cell suspensions were activated with 1 µg/ml Concanavalin A (Sigma, St. Louis, MO) at days 1 and 4 after isolation 32.

### Cell labeling and cell viability

Pmel DsRed T cells were exposed to different concentrations of ferucarbotran nanoparticles (25 µgFe/ml to 200 µgFe/ml) (Meito Sangyo Co., LTD, Japan) at day 7 after isolation for 24 h in T cell media supplemented with IL-2. After exposure, T cells were centrifuged at 500 rcf for 5 minutes at 4 °C, the supernatant was discarded, and cells were washed 3× with PBS by repeated centrifugation. Cells were resuspended in fresh T cell media. Cell viability was evaluated by two methods. DsRed fluorescence was measured in a plate reader (SpectraMax M5 Microplate Reader**,** Molecular Devices, LLC, San Jose, CA, USA) at 540 nm Ex/590 nm Em for the different groups of labeled cells. Trypan blue exclusion assay was used to quantify live cells after labeling. Data were normalized relative to the control group.

### Flow cytometry

T cells were washed with PBS containing 2% FBS prior to the addition of antibodies. Cells were stained with fluorophore-conjugated antibodies specific for mouse CD3-AF700 (BD Biosciences, San Jose, CA, USA), CD8-APC (eBioscience, Waltham, MA), CD27-PE (BD Biosciences, San Jose, CA, USA), and CD44-BV421 (BD Biosciences, San Jose, CA, USA) in PBS with 2% FBS for 15 min at 4 °C. Cells were then fixed in 4% paraformaldehyde for 10 min at 4 °C and washed twice in PBS with 2% FBS. Flow cytometry was performed on a Canto II flow cytometry system (BD Biosciences, San Jose, CA) 32.

### Functional assays

To determine if T cells remain functional after labeling with ferucarbotran, pmel DsRed T cells were co-cultured without IL-2 at a 5:1 ratio with KR158B-Luc gp100 glioblastoma cell line for 24 h. Levels of luciferase production from glioblastoma cells were measured using D-luciferin (150 µg/ml final concentration, Perkin Elmer, Waltham, MA, USA) and luminescence measurement in a plate reader after 15 minutes. T cell release of interferon gamma (IFN-γ) was evaluated after labeling. For this experiment, cells were co-cultured at the same conditions stated above in a round-bottom 96-well plate, after 24 h supernatant was collected by centrifuging the plates at 200 rcf for 5 minutes at 4 °C. Mouse IFN-γ Platinum ELISA (Invitrogen, Carlsbad, CA, USA) was performed on the supernatants 32.

### Microscopy

Labeled and unlabeled T cells were air-dried in a microscopy slide, followed by fixation in 4% paraformaldehyde and staining with Prussian Blue (Sigma, St. Louis, MO, USA) to image ferucarbotran deposits within the cell via optical microscopy. Fluorescence microscopy (Keyence Epi-Fluorescence microscope BZ-X710, Keyence, Japan) was performed using anti-dextran FITC antibody (Stemcell Technologies, Vancouver, Canada) co-incubated with T cells for 24 h in a chamber slide for live-cell imaging. Cells were stained with Hoechst 33342 for 5 minutes followed by live-cell imaging microscopy. To further evaluate ferucarbotran uptake, transmission electron microscopy of labeled T cells was performed. Cells were fixed for 24 h in a mixture of 4% glutaraldehyde and 1% paraformaldehyde. Then the sample was processed through a serial step dehydration process with ethanol, stained with osmium, and embedded in epoxy. Blocks were sectioned into 70 nm thin slices using a Leica Ultracut UCT ultramicrotome (Leica Microsystems, Wetzlar, Germany). Thin sectioned slices were dried in a copper grid prior to imaging in Hitachi H7600 electron microscope (Hitachi High-Technologies Corporation, Japan) and FIB - FEI Helios EDAX EDS/EBSD (Field Electron and Ion Company, Hillsboro, Oregon, USA).

### Iron Quantification

Two methodologies were used to quantify iron uptake: 1,10-phenanthroline assay and MPI. For the 1,10-phenanthroline colorimetric assay, 2x10^6^ cells were digested in nitric acid at a concentration of 5x10^5^ cells/ml at 115 °C until nitric fully evaporated. The sample crust that is formed was dissolved in water and reacted with hydroxylamine for 1 h, followed by the addition of sodium acetate and 1,10-phenanthroline. A calibration curve was prepared alongside the samples and absorbance was measured at 508 nm. For MPI iron quantification, 2x10^6^ cells were centrifuged in a 0.2 mL centrifuged tube and the pellet was immobilized with 4% agar solution prior to measurement in the MPI. Three fiducials containing approximately 100%, 50% and 10% of the total MPI signal were used within the same field of view (FOV) on the MPI. Longitudinal isotropic scans were taken using the Momentum^TM^ imager from Magnetic Insight (Alameda, CA, USA). Images were analyzed using VivoQuant^TM^ software, and iron uptake was determined using the known iron mass in the fiducials.

### Brain tumor implantation and ACT

Murine KR158B-Luc-gp100 glioblastoma cells were cultured as adherent monolayers and harvested with 0.05% trypsin. Tumor cells were resuspended in 1× PBS and mixed with methylcellulose (1:1 ratio) (R&D Systems). 8- to 10- week old naïve C57BL/6 mice were anesthetized with isoflurane and placed in a stereotactic frame. Intracranial implantation of 10^4^ KR158B-Luc-gp100 glioblastoma cells in 2.5 µL was performed 2 mm to the right of the bregma and 4 mm below the skull using a 25G needle attached to a 250 µL syringe (Hamilton, Reno, NV). Mice were monitored and sacrificed before reaching endpoint 32. Ferucarbotran-labeled activated pmel DsRed T cells (10^7^ cells per mouse), were washed and resuspended in 100 µL PBS, and then injected intravenously via the tail vein. For intracerebroventricular injections, T cells (10^6^ per mouse) were suspended in 5 µL PBS. Mice were anesthetized with isoflurane and placed in a stereotactic frame. 5x10^5^ cells were implanted at ±1 mm mediolateral, -0.3 mm anteroposterior, and -3 mm dorsoventral, in a volume of 2.5 µL in each of the two lateral ventricles. To improve engraftment of T cells, animals were lympho-depleted with total body irradiation using 500 rad (5 Gy) 24 h prior ACT.

### *In vivo* and *ex vivo* imaging

Mice were anesthetized with isoflurane (Patterson Companies, Inc, St. Paul, MN, USA), and 150 mg Luciferin/kg body weight were injected in the intraperitoneal cavity. Animal heads were shaved prior to imaging to avoid significant scattering/blocking of the optical signal. Animals were placed on the warmed *In vivo* Imaging System (IVIS® Spectrum CT, Perkin Elmer, Waltham, MA, USA) stage under 1.5% isoflurane and luminescence acquisition was taken 15 minutes after injection. Epi-fluorescence images using DsRed (570/620 nm) were also obtained. After IVIS®, the mouse was transferred to the MPI bed under anesthesia, and images were acquired using high sensitivity mode (3 T/m) in a FOV of 6 x 12 cm. Fiducials of known iron mass were within the same FOV in the MPI. The MPI bed was adapted to fit the CT stage to perform dual 3D imaging and anatomical co-registrations. Anatomic CT (IVIS® Spectrum CT, Perkin Elmer, Waltham, MA, USA) reference images were acquired on anesthetized animals (20 ms exposure time, 440 AI X-Ray filter). After *in vivo* imaging, mice were euthanized with isoflurane overdose and the liver, lungs, spleen, kidneys, and brain were collected and imaged via MPI and IVIS® epifluorescence.

### Histology

Immunohistochemistry of paraffin embedded, formalin fixed tissue sections of 4 µm thickness was performed. The sections were steamed for 30 minutes in a solution of citrate buffer for antigen retrieval. The primary antibodies were polyclonal rabbit anti-RFP (DsRed, PM005, MBL International), monoclonal rabbit anti-CD3 (ab16669, Abcam), mouse monoclonal anti-dextran FITC (60026Fl, Stemcell Technologies). As secondary antibody we used Cy3 donkey anti-rabbit IgG (711-165-152, Jackson ImmunoResearch Lab). Immunohistochemistry for CD3 and anti-RFP (DsRed) was performed in separate sections. Anti-RFP staining was needed because the tissue fixation and processing quenched the inherent DsRed signal from pmel DsRed T cells. Prussian Blue (Sigma, St. Louis, MO, USA) staining was also used to image iron oxide deposits within the brain tumor tissue. Fluorescence microscopy (Keyence Epi-Fluorescence microscope BZ-X710) was used for imaging.

### Image processing and analysis

DICOM^®^ files from the Momentum^TM^ were analyzed using various approaches. MPI images from *in vitro* experiments were analyzed using a MATLAB^®^ code. In order to visually compare images, a MATLAB^®^ code was written to rescale images to their original representation. The rescale slope tag in the DICOM® was used to specify a linear transformation from pixels. Areas of analysis were defined by the interactive positioning of rectangle masks in the regions to be analyzed. Here, a region grow algorithm was set to determine a threshold of 50-80% of the maximum signal intensity to define a region of interest (RoI). Background noise was defined as the maximum signal intensity detected in MPI blank scans from the MPI bed or sample holder where no sample was present. For *in vivo* MPI, optical photos and 2D MPI images were co-registered with fiducials using VivoQuant^TM^ (Invicro, LLC, Boston, MA, USA), and 3D MPI and CT-scan images were co-registered using 3D Slicer open-source software 46. *In vivo* MPI signal quantification was performed using the threshold function in 3D Slicer. IVIS^®^ luminescence and epi-fluorescence images were analyzed using Living Image^®^ software (Perkin Elmer, Waltham, MA, USA).

### Statistical analysis

One-way ANOVA with Tukey's multiple comparison test were performed to determine statistical significance for *in vitro* experiments. Statistical significance difference was set for p-values less than 0.05. Graph Pad Prism (La Jolla, CA) and Microsoft Excel (Redmond, WA) were used to conduct all analyses. Error bars represent the standard error of the mean.

## Supplementary Material

Supplementary figures and tables.

## Abbreviations

ACT: adoptive cellular therapy
ANOVA: analysis of variance
CD: cluster of differentiation
CNS: central nervous system
CT: computed tomography
DICOM: digital imaging and communications in medicine
DMEM: Dulbecco's modified eagle medium
EDX: energy dispersive x-ray
FBS: fetal bovine serum
Fe: iron
FOV: field of view
gp100: glycoprotein 100
IACUC: institutional animal care and use committee
IFN- γ: interferon-gamma
IL-2: interleukin-2
IVIS: *in vivo* imaging system
Kluc-gp100: KR158B-luc-gp100
Luc: luciferase
MATLAB: matrix laboratory
MPI: magnetic particle imaging
MR: magnetic resonance
PBS: phosphate buffered saline
PET: positron emission tomography
RoI: Region of Interest
RPMI: Roswell Park Memorial Institute
SPECT: single-photon emission computed tomography
SPIO: superparamagnetic iron oxide
TEM: transmission electron microscopy
