# Supplementary material for: Tracking adoptive T cell immunotherapy using magnetic particle imaging
Source: Nanotheranostics. 2021 Apr 27;5(4):431–44. doi: 10.7150/ntno.55165 (PMC8100755; doi:10.7150/ntno.55165)
Supplement: Supplementary file 1 — Supplementary figures and tables. [file ntnov05p0431s1.pdf]

Title: Tracking adoptive T cell immunotherapy using magnetic particle imaging

Authors:

Angelie Rivera-Rodriguez,[1] Lan B. Hoang-Minh,[2,3] Andreina Chiu-Lam,[4] Nicole Sarna,[1] Leyda Marrero-Morales,[1] Duane A. Mitchell,[2,3,5] Carlos M. Rinaldi-Ramos,[1,4,5,*]

Affiliations:

[1]J Crayton Pruitt Family Department of Biomedical Engineering, University of Florida, Gainesville, FL USA
[2]Preston A. Wells, Jr. Center for Brain Tumor Therapy, University of Florida, Gainesville, FL USA
[3]Lillian S. Wells Department of Neurosurgery, McKnight Brain Institute, University of Florida, Gainesville, FL USA
[4]Department of Chemical Engineering, University of Florida, Gainesville, FL USA
[5]UF Health Cancer Center, University of Florida, Gainesville, FL USA
*Corresponding author. Email: carlos.rinaldi@ufl.edu

Supplementary Materials

MPI Limit of Detection: To determine the limit of detection (LoD) in our MPI system, a dilution series of ferucarbotran tracer was prepared, with decreasing iron mass by a factor of 2. All scanned samples consisted of a volume of 1 µL in a 0.2 mL microcentrifuge tube. Samples (n=3) were imaged at two different positions within the MPI field of view. MPI scans were performed using the Momentum™ imager from Magnetic Insight (Alameda, CA, USA) in the high-sensitivity (3 T/m) scan mode. The images were analyzed using VivoQuant™ and MATLAB® to evaluate signal intensity in regions of interest containing the tracer samples. The LoD was calculated by analyzing the background signal from empty scans, the limit of blank (LoB), and the maximum intensity signal of samples at low concentrations [47].

$$LoB = mean_{blank} + 1.645(SD_{blank})$$

$$LoD = LoB + 1.645(SD_{low\ concentration\ sample})$$

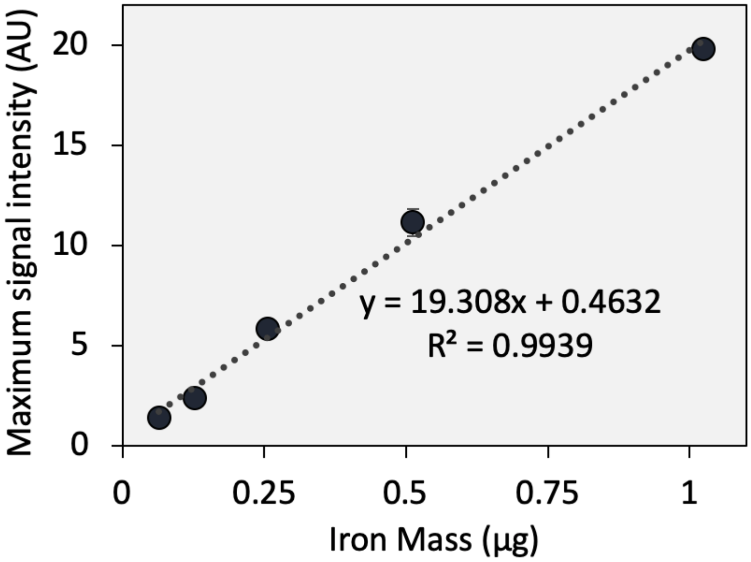

Fig. S1. Linear correlation of ferucarbotran iron mass and MPI signal intensity. Note: Error bars are smaller than the markers.

Table S1. Values used to determine the limit of detection (LoD) for ferucarbotran.

	MPI signal
$Mean_{Blank}$	-0.013
SD_{Blank}	0.269
LoB	0.429
$SD_{Max\ Signal\ at\ 64\ ngFe}$	0.108
LoD	0.608

For the dilution series of ferucarbotran, the signal intensity decreases with decreasing iron mass (**Fig. S1)**. The high-sensitivity scan mode used throughout this paper has a low gradient field strength of 3 T/m. Low gradient fields do not fully saturate the particles in the vicinity of the field free region, and more signal can be obtained at the expense of resolution. The calculated limit of detection is approximately 38 ng of iron for ferucarbotran nanoparticles, which is consistent with the lowest number of ferucarbotran-labeled T cells (~50,000 cells) that were detect *in vitro*. The LoD in terms of iron mass was calculated using the LoD in signal intensity from **Table S1**, and the value was correlated to iron mass using the regression line from **Fig. S1**.
